# Precise Investigation of the Efficacy of Multicomponent Drugs Against Pneumonia Infected With Influenza Virus

**DOI:** 10.3389/fphar.2021.604009

**Published:** 2021-11-18

**Authors:** Junying Wei, Jianhui Sun, Jiawei Zeng, Enhui Ji, Jing Xu, Chunyu Tang, Hairu Huo, Yi Zhang, Hongmei Li, Hongjun Yang

**Affiliations:** ^1^ Institute of Chinese Materia Medica, China Academy of Chinese Medical Sciences, Beijing, China; ^2^ Department of Clinical Laboratory, Mianyang Central Hospital, Mianyang, China; ^3^ Research Center of Anti-infection Chinese Medicine Engineering Technology, Yongzhou, China

**Keywords:** pneumonia, precise medicine, multicomponent drug, proteomics, houyanqing oral liquid

## Abstract

**Background:** Viral pneumonia is one of the most serious respiratory diseases, and multicomponent traditional Chinese medicines have been applied in the management of infected patients. As a representative TCM, HouYanQing (HYQ) oral liquid shows antiviral activity. However, the unclear mechanisms, as well as the ambiguous clinical effects, limit widespread application of this treatment. Therefore, in this study, a proteomics-based approach was utilized to precisely investigate its efficacy.

**Methods:** Based on the efficacy evaluation of HYQ in a mouse model of pneumonia caused by influenza A virus (H1N1) and the subsequent proteomics analysis, specific signatures regulated by HYQ treatment of viral pneumonia were identified.

**Results:** Experimental verifications indicate that HYQ may show distinctive effects in viral pneumonia patients, such as elevated galectin-3-binding protein and glutathione peroxidase 3 levels.

**Conclusion:** This study provides a precise investigation of the efficacy of a multicomponent drug against viral pneumonia and offers a promising alternative for personalized management of viral pneumonia.

## 1 Introduction

Pneumonia is one of the most serious respiratory diseases, causes many deaths per year and is often caused by viruses, especially severe acute respiratory syndrome (SARS) virus (Levy et al., 2005), the 2009 pandemic influenza A (H1N1) virus (Writing Committee of the WHO Consultation on Clinical Aspects of Pandemic (H1N1) 2009 Influenza et al., 2010) and coronavirus disease 2019 (COVID-19) (Guan et al., 2020). Along with the increase in our understanding of the role of viruses in pneumonia, more findings indicate that the incidence of viral pneumonia has been underestimated (Ruuskanen et al., 2011). Thus, further studies are urgently needed that focus on the cause and pathogenesis of viral pneumonia, better management and care of patients, and identification of safer and more effective drugs, including personalized medicine for viral pneumonia patients (Schuetz et al., 2013; Rello and Perez, 2016; Gautam et al., 2018).

Multicomponent drugs represented by traditional Chinese medicine (TCM) have drawn increasing attention in viral pneumonia treatment (Wang et al., 2011; Li et al., 2015; Zhu et al., 2018; Hu et al., 2020; Li et al., 2020; Yang et al., 2020). As a typical representative, Lianhuaqingwen can affect virus morphology and exert anti-inflammatory activity *in vitro* (Li et al., 2020). Moreover, a multicenter open-label randomized controlled trial indicated that the recovery rate of patients with COVID-19 was higher in the Lianhuaqingwen treatment group than in the control group, and the median time to recovery was also shorter in the treatment group (Hu et al., 2020). Although these TCMs have shown better effects and fewer side effects in some cases, due to the characteristics of TCMs, such as multiple components and superposition effects, further investigations are needed to reveal their potential mechanisms and precise effects in the clinic (Wei et al., 2019a; Wei et al., 2019b).

In this study, we utilized a proteomics-based approach to precisely investigate the efficacy of a multicomponent drug, HouYanQing (HYQ) oral liquid, against pneumonia due to influenza virus in mice. As a traditional local medicine in Hunan Province of China, HYQ contains 4 types of herbs, *Achyranthes aspera* L. *Aster indicus* L. *Carpesium abrotanoides* L. and *Plantago depressa* Willd*.* in a ratio of 250:143:36:71 (Chinese Pharmacopoeia 2020). A previous study indicated that HYQ can decrease the viral load in the lungs of mice with pneumonia due to influenza virus and plays a therapeutic role in influenza A virus infection in mice (Sun et al., 2019). However, the corresponding mechanism, as well as its potential clinical application in personalized medical care of viral pneumonia, merits further investigation. This study provides a precise investigation of the efficacy of multicomponent drugs against viral pneumonia and offers a promising alternative for personalized management of viral pneumonia.

## 2 Materials and Methods

### 2.1 Drugs and Materials

HouYanQing (HYQ) oral liquid extracts were obtained from Hunan Time Sun Pharmaceutical Co., Ltd. (Yongzhou, China. Batch 2018010908). Oseltamivir phosphate capsules were purchased from Shanghai Roche Pharmaceutical Ltd. (Shanghai, China. Batch H1035). Sequencing grade porcine trypsin and dithiothreitol (DTT) were obtained from Promega (Madison, WI, United States ). Iodoacetamide (IAA) was purchased from Sigma-Aldrich Chemicals (St. Louis, Missouri, United States ). All of the other chemicals were analytical grade reagents. Deionized water (R > 18.2 MΩ) used for all of the experiments was purified by using a Millipore purification system (Billerica, MA, United States ).

The content of HYQ was determined based on Chinese Pharmacopoeia 2020. The sample was hydrolyzed with hydrochloric acid-ethanol for 1 h and extracted with petroleum ether (60–90°C). HPLC determinations were performed by using a SHIMADZU LC-20AT instrument (Shimadzu Corporation, Japan) equipped with Diamonsil C18 (250 mm × 4.6 mm, 5 μm) column. The mobile phase was acetonitrile (A)-Water (B) (90:10), the detection wavelength was 210 nm, the column temperature was 30 °C and the flow rate was 1 ml/min.

### 2.2 Animal Study

Specific-pathogen-free (SPF) male ICR mice (Charles River Laboratories, SCXK 2016–0006), 13–15 g in weight, were mildly anesthetized with ether and then infected by nasal administration of the type A influenza virus FM_1_ mouse lung-adapted strain (obtained from Institute of Virology, Chinese Academy of Preventive Medicine) at a concentration of *15 LD50* (each 0.05 ml). From the day of virus infection, the infected mice were randomly divided into the treatment groups and administered high-dosage HYQ (15.6 g/kg daily, *n* = 24), low-dosage HYQ (3.9 g/kg daily, equivalent to a clinical dosage, *n* = 24), oseltamivir (40 mg/kg daily, *n* = 24) or water (model, *n* = 24) by oral administration (p.o.) twice per day for nine consecutive days. Uninfected mice were used as controls (*n* = 24). Both infected animals and control animals were euthanized three, six, and 9 days postinfection (eight mice were euthanized in each group at each time point). Plasma samples were collected and frozen at-20°C, and lung tissues were harvested and frozen at-80°C for the next analysis. The lung index was calculated as follows: lung index = lung weight (g)/body weight (g) *100%. All of the animal experiments were approved by the Committee on the Animal Care and Use of the Institute of Chinese Materia Medica, China Academy of Chinese Medical Sciences and were carried out in accordance with the approved guidelines. All viral experiments were performed in the ABSL-2 biosafety room.

### 2.3 Determination of Viral Load in Lung Tissue by Real-Time PCR (RT-PCR)

The viral load in the lung tissue of an infected mouse was determined by RT-PCR. The primers of FM_1_ for RT-PCR were as follows: upstream, 5′-AAA​CCC​AGA​ATG​CGA​ATC​AC-3'; downstream, 5′-GCT​CAG​CTT​TGG​GTA​TGA​GC-3' (synthesized by Invitrogen). Briefly, total RNA was extracted using TRIzol (TaKaRa, Japan. Batch R6321) according to the manufacturer’s instructions, and reverse transcription reactions were performed using the PrimeScript™ RT reagent kit (TaKaRa, Japan. Batch AK3001). The RT-PCR protocol was as follows: 95°C, 30 s; 95°C, 5 s; 60°C, 40 s; with 40 amplification cycles; 95°C, 10 s using the ABI 7500 RT-PCR machine. The assays were carried out in triplicate, and Ct values were calculated. The copy number was calculated according to the standard curve.

### 2.4 Proteomic Analysis

Mouse lung tissues were homogenized in PBS (KCl: 0.2 g, KH_2_PO_4_: 0.2 g, NaCl: 8.0 g, Na_2_HPO_4_.12H_2_O: 3.9054 g, pH 7.4, 1000 ml) buffer cocktail with a tissue homogenizer (Sceintz-48, Sceintz, Ningbo, China). Then, the proteins of mouse lung tissues were lysed with 8 M urea by ultrasound, and the lysate was centrifuged at 24,000 g for 30 min at 4°C. The supernatant was collected, and the protein concentration was determined by the bicinchoninic acid assay. Three hundred micrograms of protein was reduced by adding 0.1 M dithiothreitol for 4 h at 37°C and then alkylated by adding 0.5 M IAA for 60 min at room temperature in the dark. The protein sample was finally digested using trypsin in 50 mM ammonium bicarbonate (pH 8.0) at an enzyme:protein mass ratio of 1:50 for 24 h at 37°C.

Orbitrap Fusion (Thermo Fisher Scientific) LC-MS/MS analyses were performed on an Easy-nLC 1000 liquid chromatography system (Thermo Fisher Scientific) coupled to an Orbitrap Fusion via a nanoelectrospray ion source. Tryptic peptides were dissolved with loading buffer (acetonitrile and 0.1% formic acid), and the tryptic peptides were eluted from a 150 μm ID x 2 cm C18 trap column and separated on a homemade 150 µm ID x 12 cm column (C18, 1.9 μm, 120 Å, Dr Maisch GmbH) with a flow rate of 500 nL/min. Survey scans were acquired after an accumulation of 5e^5^ ions in the Orbitrap for m/z 300–1,400 using a resolution of 120,000 at m/z 200. The top speed data-dependent mode was selected for fragmentation in the HCD cell at a normalized collision energy of 32%, and fragment ions were then transferred into the ion trap analyzer with the AGC target at 5e^3^ and maximum injection time at 35 m. The dynamic exclusion of previously acquired precursor ions was enabled at 18 s. Spectral data were searched against the mouse protein RefSeq database (downloaded on 9–26–2018) in Proteome Discoverer1.4.1.14 suites with Mascot software (version 2.3.01, Matrix Science) to achieve a false discovery rate of <1%. The mass tolerance was set at 10 ppm for precursor ions, and it was set at 0.5 Da for the tolerance of product ions. Oxidation (M) and acetylation (Protein-N term) were chosen as variable modifications, while carbamidomethylation (C) was chosen as a fixed modification, and two missed cleavage sites for trypsin were allowed.

Intensity-based absolute quantification (iBAQ)-based protein quantification (Schwanhäusser et al., 2011; Wei et al., 2019b) was performed using in-house software. Briefly, the iBAQ intensities were obtained by dividing the protein intensities by the number of theoretical peptides, which were calculated by *in silico* protein digestion with a PERL script, and all of the fully tryptic peptides between 6 and 30 amino acids were counted, while the missed cleavages were neglected. The iBAQ value of each protein was then normalized to the total iBAQ value for all of the identified proteins to avoid possible experimental variations (Wei et al., 2014; Wei et al., 2016). Three individual samples were analyzed in each group, and the changes were identified by statistical analyses of the measured protein amounts from each individual sample (Wei et al., 2014).

### 2.5 Western Blot Analysis

The proteins of mouse lung tissues were extracted in ice-cold RIPA lysis buffer (Solarbio, China) by ultrasound and then determined by the enhanced bicinchoninic acid protein assay kit (Thermo, United States ). Thirty micrograms of each sample was loaded on 10% SDS-PAGE gels, and protein blots were transferred onto polyvinylidene fluoride membranes (Millipore, United States ). After blocking with 5% nonfat milk, the blots were incubated overnight at 4°C with the following primary antibodies: anti-glyceraldehyde-3-phosphate dehydrogenase (GAPDH, Proteintech, 10494-1-AP), anti-intercellular adhesion molecule-1 (ICAM-1, Proteintech, 16174-1-AP) and anti-transferrin (Proteintech, 17435-1-AP). Then, the membranes were washed with a mixture of Tris-buffered saline and Tween 20 (TBST) and incubated at room temperature for 1 h with a secondary antibody conjugated to horseradish peroxidase. Finally, the protein blots were visualized using an enhanced chemiluminescence kit (Millipore, United States ). Three individual samples were analyzed in each group. Bar graphs are relative gray values to GAPDH. The results from each mouse were analyzed by one-way analysis of variance.

### 2.6 ELISA

Serum samples from the mice were centrifuged at 10,000 rpm for 10 min at 4°C. The supernatant was assayed using ELISA kits according to the manufacturer’s instructions. Mouse galectin-3-binding protein (*Lgals3bp,* Cusabio, CSB-EL012888MO), mouse plasma protease C1 inhibitor (*Serping1,* Cusabio, CSB-EL021086MO), mouse glutathione peroxidase 3 (*Gpx3,* Cusabio, CSB-EL009868MO), mouse hemopexin (*Hpx,* Abcam, ab157716) and protein S100-A8 (*S100a8,* Abcam, ab263886) ELISA kits were used. Each kit consisted of a 96-well plate in which a specific antibody against a target protein was immobilized. The target protein in sera is recognized by the antibody, followed by incubation with a horseradish peroxidase-conjugated secondary antibody for colorimetric quantification. The plates were read on a microplate reader (Molecular Devices, United States ) at 450 nm. The reactions were carried out in triplicate for each sample. Finally, the results were analyzed by one-way analysis of variance and were considered significant at *p <* 0.05.

## 3 Results

### 3.1 Protective Effect of HYQ on the Mouse Model of Pneumonia Infected With Influenza Virus

**FIGURE 1:**
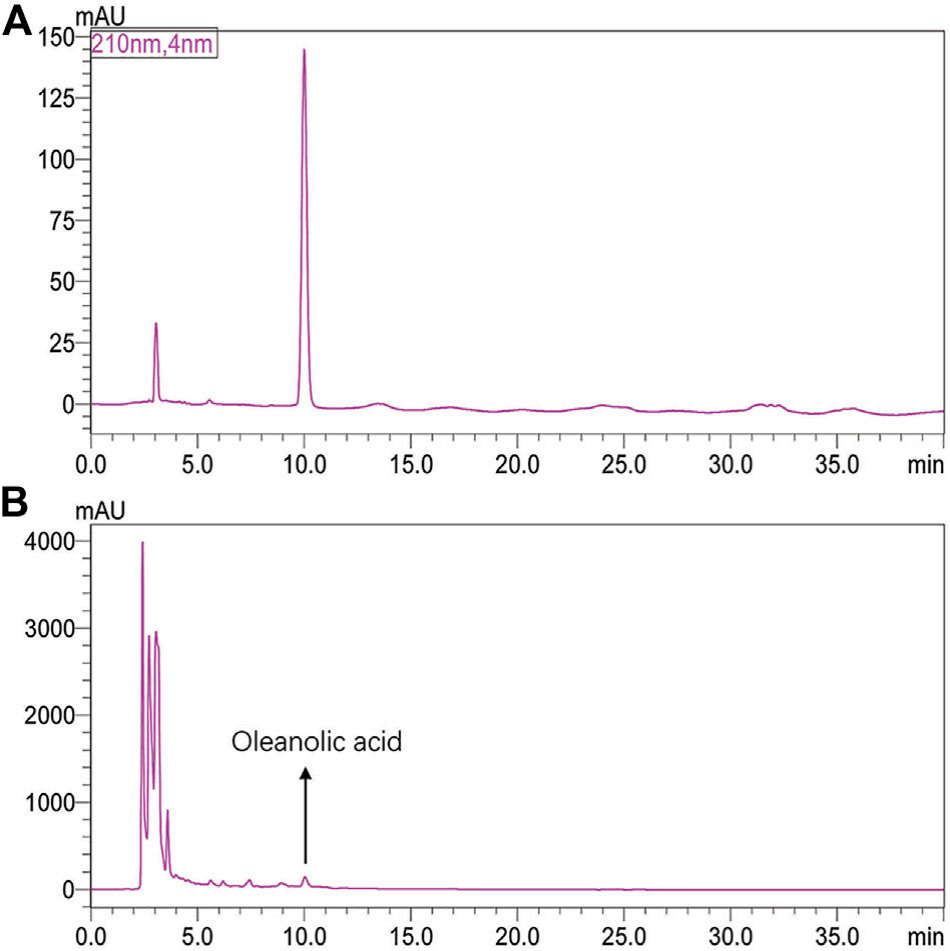
HPLC chromatograms of the standard oleanolic acid **(A)** and HYQ Sample **(B)**.

**FIGURE 2:**
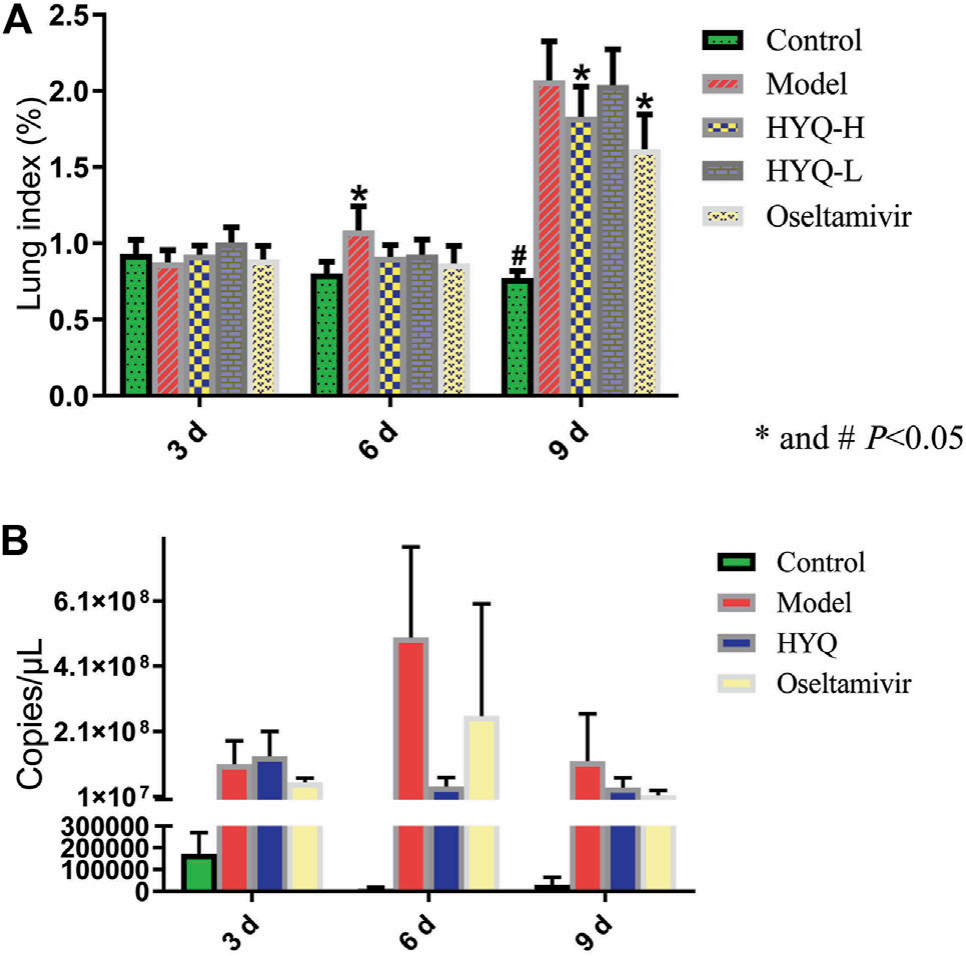
Efficacy evaluation of HYQ against viral pneumonia in mice infected with influenza A virus (H1N1) **(A)** Lung index. HYQ-H: high-dosage HYQ. HYQ-L: low-dosage HYQ **(B)** Viral load results of the mouse lung. HYQ: high-dosage HYQ. 3, 6 and 9 days: virus infection time.

First, the content of HYQ was determined based on Chinese Pharmacopoeia 2020. As shown in Figure 1, the retention time was 10.007 min for oleanolic acid and the content was 4.57 mg/g, which is in accord with Chinese Pharmacopoeia 2020. Then, the therapeutic effect of HYQ on the mouse model of pneumonia infected with influenza A virus (H1N1) was investigated at the third, sixth, and ninth days after influenza virus infection. As shown in Figure 2A, along with prolongation of virus infection, the lung index of the infected mice was increasingly higher than that of the control mice. At the sixth day after infection, both HYQ and oseltamivir decreased the lung index of the infected mice, and at the ninth day, the high dose of HYQ and oseltamivir remained effective. The viral load in the lung tissues of the infected mice was significantly higher than that of the other mice on the sixth day after infection (Figure 2B), and both high-dose HYQ and oseltamivir reduced the viral load of the infected mice, even on the ninth day. These experimental results indicate that after almost 1 week of intervention, HYQ can exert therapeutic efficacy in mice with pneumonia due to influenza virus.

### 3.2 Investigation of the Protein Dynamics of the HYQ-Mediated Effects on Viral Pneumonia in Mice

**FIGURE 3:**
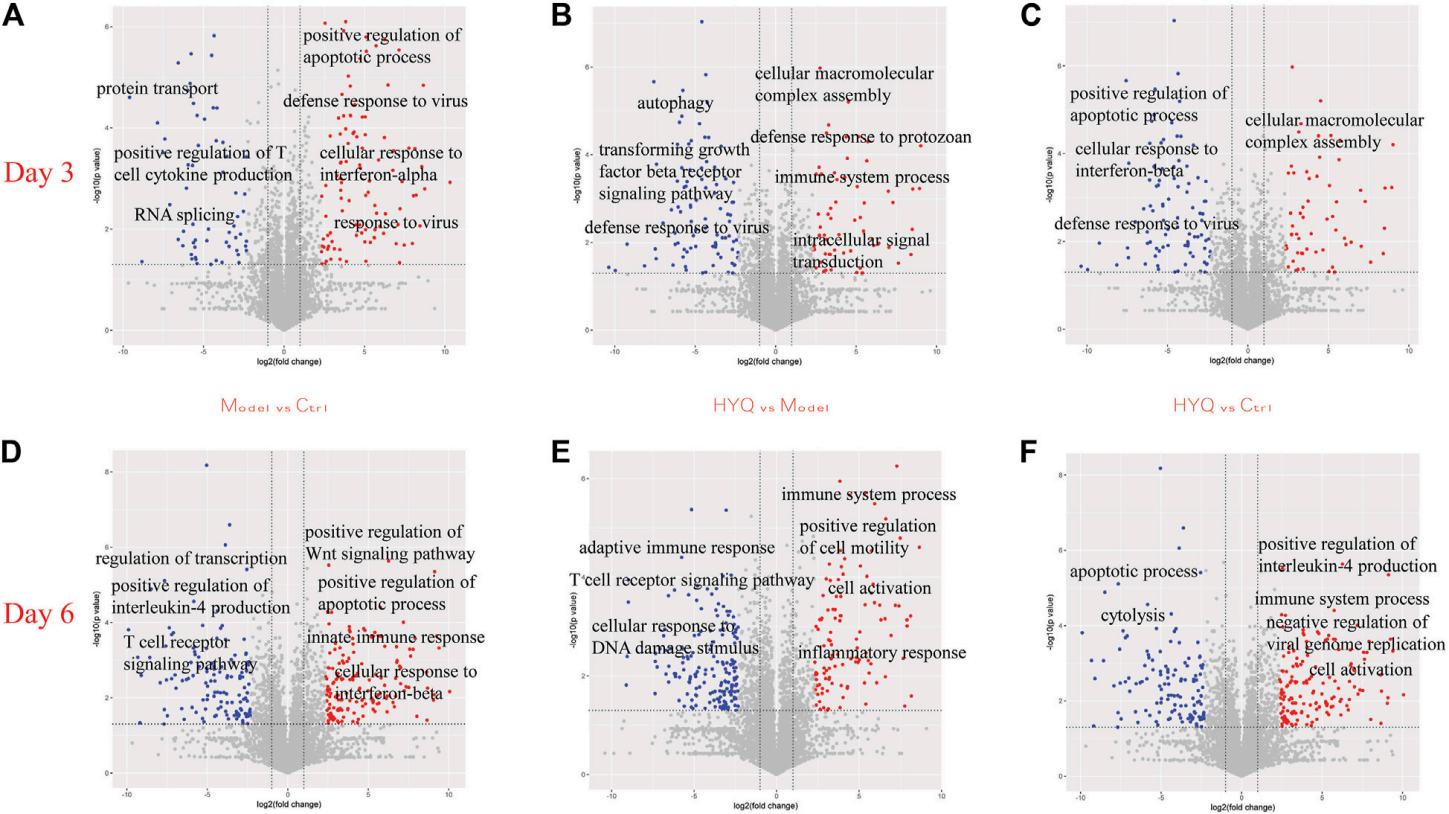
Protein dynamics of HYQ-mediated intervention of viral pneumonia in the mouse lung at the third and sixth days after viral infection and high-dosage HYQ administration **(A)** Proteins’ change at the third day after viral infection (model vs control at day 3) and the biological process or molecular function of some proteins with significantly different expression levels (*p* < 0.05) **(B)** Proteins’ change at the third day after viral infection and high-dosage HYQ administration (HYQ vs model at day 3) and the biological process or molecular function of some proteins with significantly different expression levels (*p* < 0.05) **(C)** Proteins’ change between high-dosage HYQ administration and control at the third day (HYQ vs control at day 3) **(D**) Model vs control at day 6 **(E)** HYQ vs model at day 6 **(F)** HYQ vs control at day 6. Ctrl: control. Days 3 and 6: virus infection time.

The protein dynamics following HYQ-mediated intervention of viral pneumonia were investigated by measuring the changes in the proteins in the mouse lung at the third and sixth days after viral infection and high-dosage HYQ administration using a high-throughput proteomics method and an accurate intensity-based absolute quantification (iBAQ)-based quantification approach (Schwanhäusser et al., 2011; Wei et al., 2014; Wei et al., 2016; Wei et al., 2019a; Wei et al., 2019b). Moreover, the iBAQ value of a protein was normalized by the total iBAQ value for all of the identified proteins to avoid possible experimental variations (Wei et al., 2014; Wei et al., 2016; Wei et al., 2019a; Wei et al., 2019b), and in each group, three individual samples were analyzed. In total, 6,208 proteins were identified (Supplementary Table S1). As shown in Figure 3A, at the third day after viral infection, compared with those in the control group, some proteins in the model group with functions such as positive regulation of apoptotic process, defense response to virus and cellular response to interferon-alpha were upregulated (*p* < 0.05), while some proteins that function in protein transport, positive regulation of T cell cytokine production and RNA splicing were downregulated (*p* < 0.05). After intervention with HYQ (Figure 3B), some proteins that are associated with immune system processes, defense responses to protozoans, intracellular signal transduction and others had upregulated expression levels (*p* < 0.05), while other proteins with functions such as defense responses to viruses, transforming growth factor beta receptor signaling pathways and autophagy decreased (*p* < 0.05). Furthermore, compared with the control (Figure 3C), HYQ administration increased the proteins that function in cellular macromolecular complex assembly and others (*p* < 0.05) and decreased the proteins associated with positive regulation of apoptotic process, cellular response to interferon-beta, defense response to virus and so on (*p* < 0.05).

Six days after virus infection, compared to those in the control (Figure 3D), the proteins in the infected mice with the function of positive regulation of the Wnt signaling pathway, positive regulation of the apoptotic process and innate immune response showed upregulated expression (*p* < 0.05), while the decreased proteins are involved in regulation of transcription, positive regulation of interleukin-4 production, T cell receptor signaling pathway and others (*p* < 0.05). Administration of HYQ enhanced the proteins involved in immune system process, positive regulation of cell motility, inflammatory response and so on (*p* < 0.05, Figure 3E), and proteins associated with adaptive immune response, T cell receptor signaling pathway and response to DNA damage stimulus were downregulated (*p* < 0.05). Additionally, compared with the control (Figure 3F), six-day intervention by HYQ increased the proteins that function in positive regulation of interleukin-4 production and negative regulation of viral genome replication (*p* < 0.05) while decreasing the proteins involved in apoptotic processes and cytolysis (*p* < 0.05). Therefore, the functional changes of these proteins are consistent with the effect of the drug.

**FIGURE 4:**
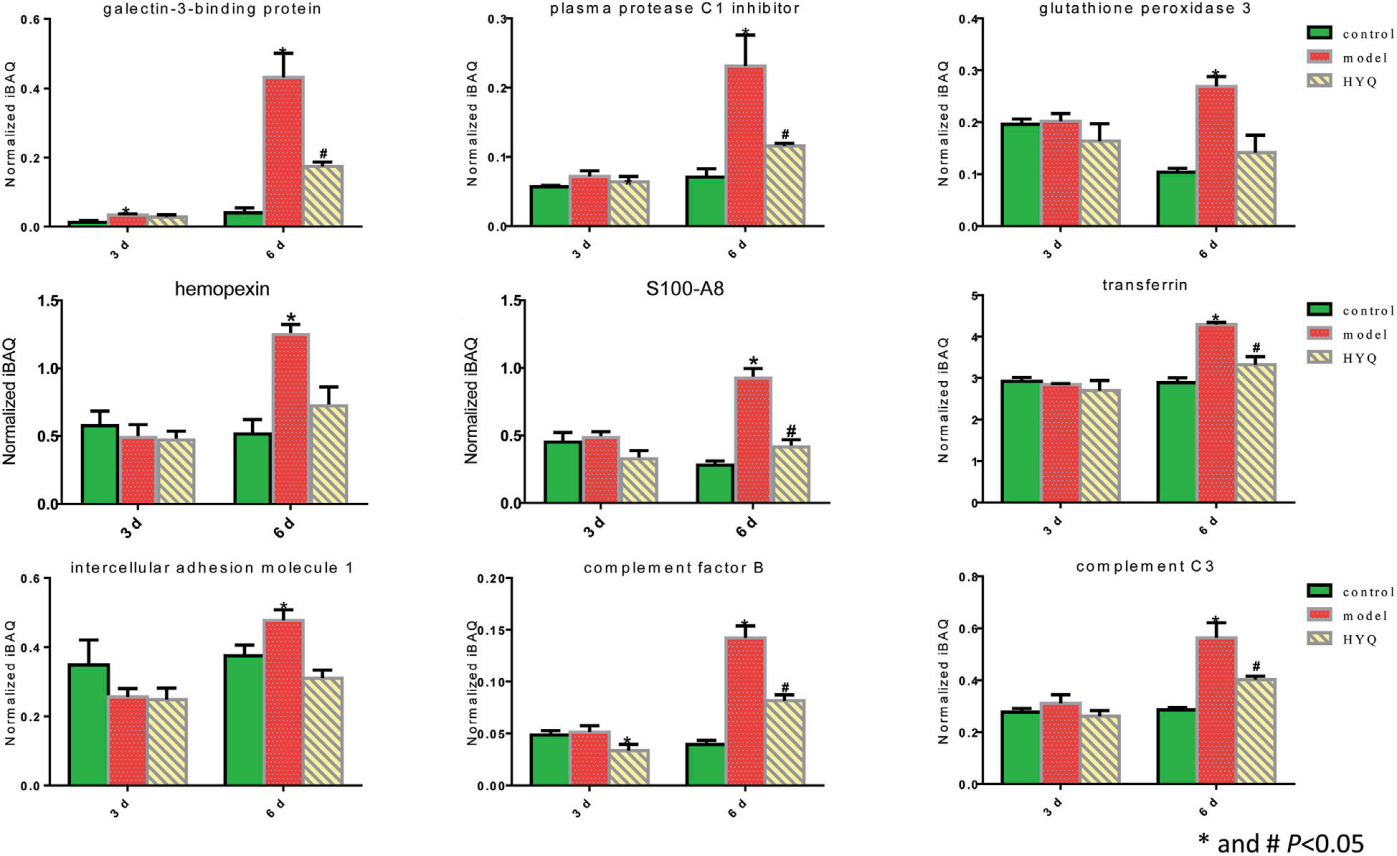
Proteomic quantitative analysis of the candidate signatures regulated by HYQ against viral pneumonia in mouse lung. Three individual samples were analyzed in each group. The normalized iBAQ value of each protein from each individual sample was used for one-way analysis of variance; 3 and 6 days: virus infection time.

Based on the statistical analyses of the measured protein amounts from each individual sample in one group (Wei et al., 2014), we investigated the dynamics of some proteins at the proteomic level. These selected proteins, such as plasma protease C1 inhibitor, hemopexin, transferrin, complement factor B and complement C3, have been identified as candidate biomarkers of acute respiratory virus infection (Burke et al., 2017) or exhibited obvious fold changes during influenza infection (Kumar et al., 2014). As shown in Figure 4, on the third day after viral infection and HYQ administration, none of these candidate proteins were significantly different among these groups. At the sixth day, compared with those of the control group, all of these candidate proteins in the infected mice had upregulated expression levels (*p* < 0.05), and administration of HYQ decreased their expression levels (*p* < 0.05). Similarly, these changes are also consistent with the effect of the drug.

### 3.3 Verification of Functional Proteins in the HYQ-Mediated Effect on Viral Pneumonia in Mice

**FIGURE 5:**
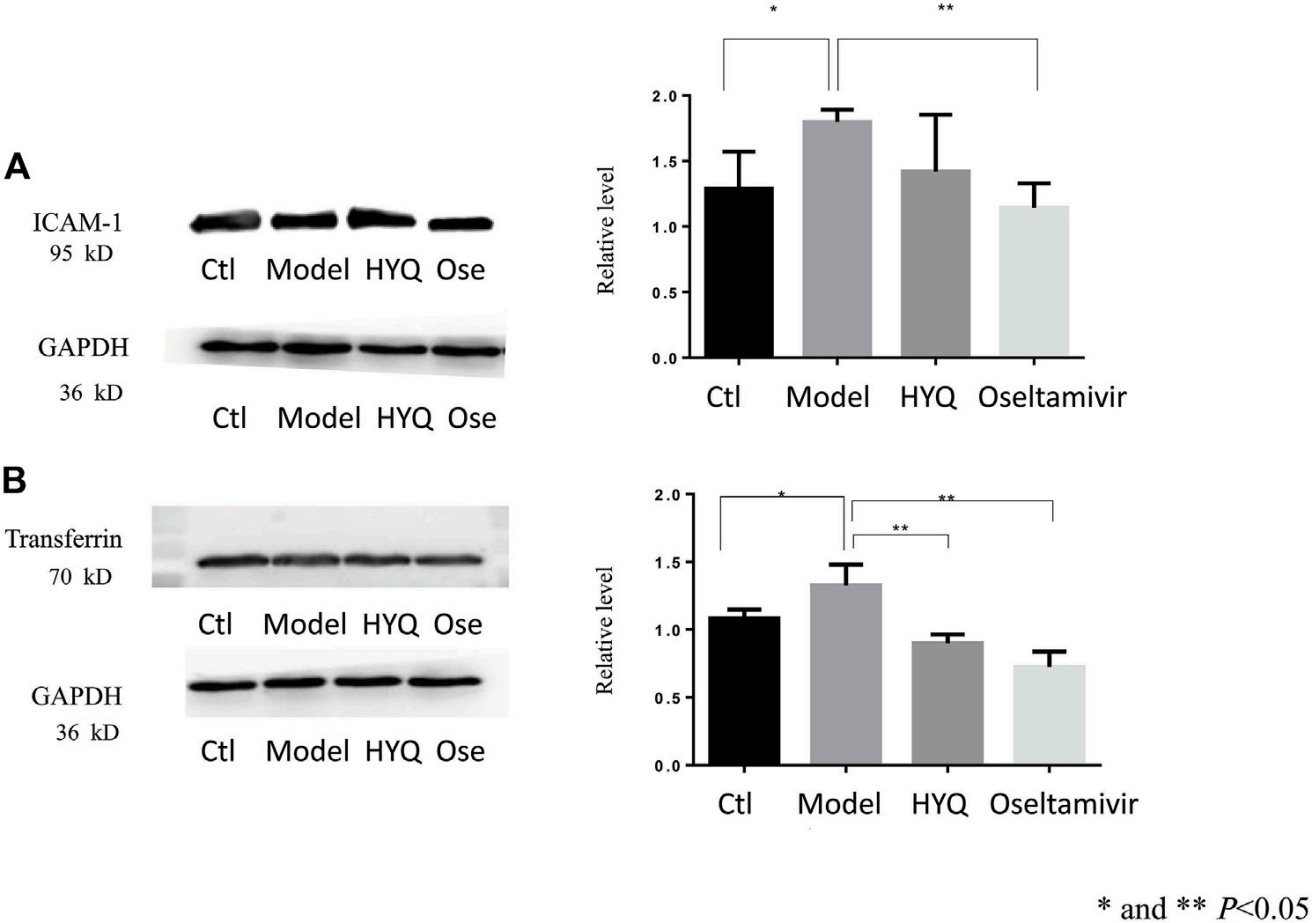
Western blot verification of functional proteins in the HYQ-mediated effect on viral pneumonia in mouse lungs at the sixth day after infection **(A)** Image of ICAM-1 and its relative level among different groups **(B)** Image of transferrin and its relative level among different groups. Bar graphs are relative gray values to GAPDH. Three individual samples were analyzed in each group, and the results from each mouse were analyzed by one-way analysis of variance. ICAM-1: intercellular adhesion molecule-1. GAPDH: glyceraldehyde-3-phosphate dehydrogenase. Ctl: control. Ose: oseltamivir.

Critical functional proteins associated with the HYQ-mediated effect on viral pneumonia in mice were verified by Western blot analysis of these proteins in mouse lung tissues at the sixth day after viral infection and HYQ administration. As shown in Figure 5, as a highly expressed protein in inflammatory conditions (Lawson and Wolf, 2009), intercellular cell adhesion molecule-1 (ICAM-1) had upregulated expression levels in the infected mice compared with the control mice (*p* < 0.05), while both HYQ and oseltamivir decreased its expression, but the HYQ administration group had no significant difference due to the substantial variation. Transferrin was shown to be influenced by inflammation and oxidative stress (Matusiewicz et al., 2017), while both HYQ and oseltamivir can reverse the upward trend in infected mice (*p* < 0.05). Thus, these critical functional proteins were verified, which indicated that HYQ may also have an anti-inflammatory effect on the infected mice.

### 3.4 Investigation of the Efficacy of HYQ on Viral Pneumonia in Mice

**FIGURE 6:**
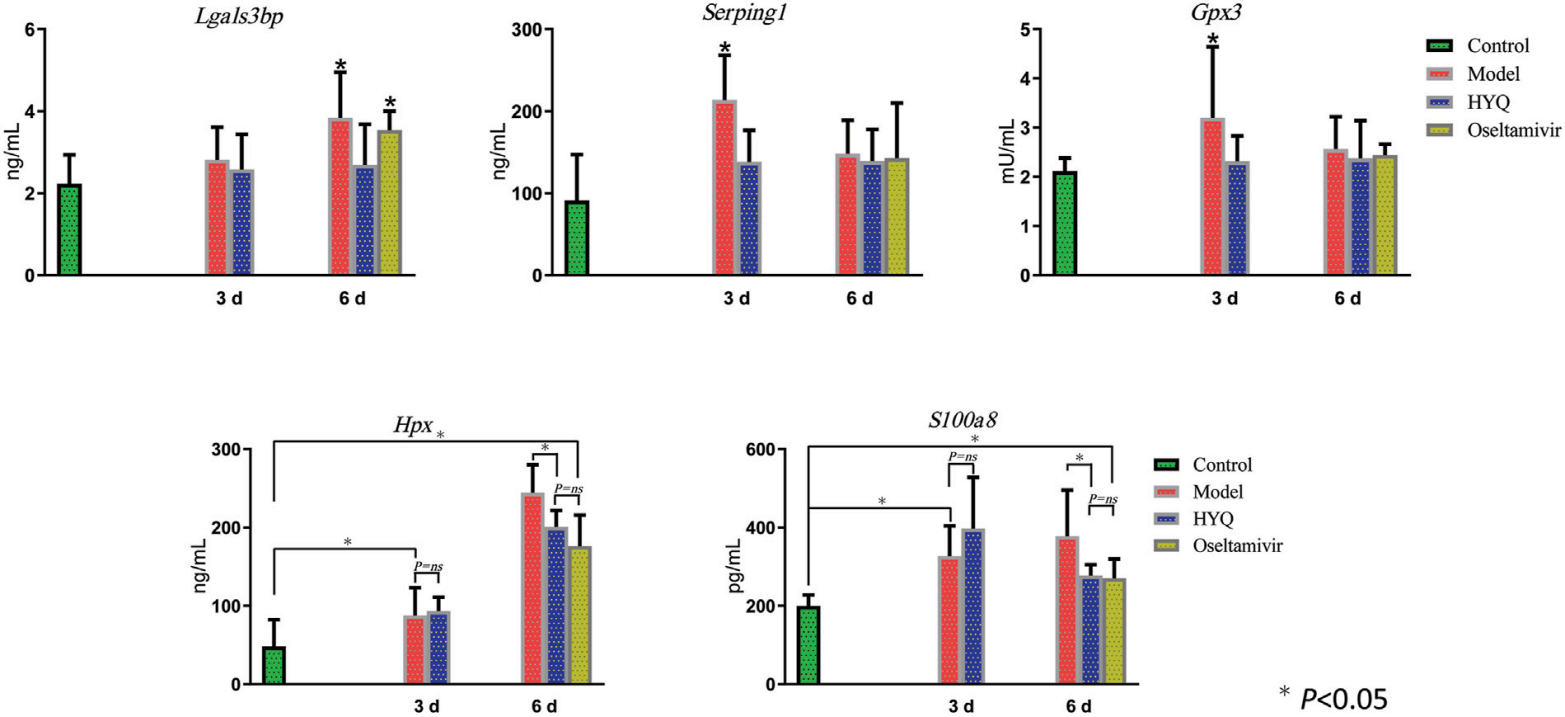
ELISA-based verification of the candidate signatures regulated by HYQ against viral pneumonia in mouse blood. Each group contained 6 mice, and the results from each mouse were analyzed by one-way analysis of variance; 3 and 6 days: virus infection time. ns: no significance. *Lgals3bp*, galectin-3-binding protein; *Serping1*, plasma protease C1 inhibitor; *Gpx3*, glutathione peroxidase 3; *Hpx*, hemopexin; *S100a8*, protein S100-A8.

Then, specific signatures related to the effects of HYQ against viral pneumonia were ultimately verified by ELISA-based measurements of the changes in the blood of the infected mice. As shown in Figure 6, on the third day after viral infection and HYQ administration, the amounts of plasma protease C1 inhibitor (*Serping1*), glutathione peroxidase 3 (*Gpx3*), hemopexin (*Hpx*) and protein S100-A8 (*S100a8*) in the infected mouse blood increased compared to that of the control (*p* < 0.05), and HYQ inhibited the elevations of *Serping1* and *Gpx3* compared with that of the model group (*p* < 0.05). On the sixth day, the amounts of galectin-3-binding protein (*Lgals3bp*), *Hpx* and *S100a8* in the infected mouse blood consistently increased during viral infection, whereas their expression levels in blood were all decreased by HYQ administration (*p* < 0.05). However, the expression level of *Lgals3bp* was not affected after oseltamivir administration (*p* > 0.05). Therefore, *Lgals3bp* is thought to correspond to the specific efficacy of HYQ against viral pneumonia. Moreover, oseltamivir was reported to have a potential side effect on the hepatic activities of glutathione reductase, glutathione peroxidase, and glutathione S-transferase (El-Sayed and Al-Kahtani, 2011). Thus, in some cases, HYQ may be a promising alternative treatment for viral pneumonia, and *Gpx3* may be another specific signature regulated by HYQ against viral pneumonia. Overall, HYQ may have a specific effect on viral pneumonia such as elevated *Lgals3bp* and *Gpx3* levels in the blood.

## 4 Discussion

It was reported that a significant number of pneumonia cases are caused by viruses (Guan et al., 2020); thus, safe and effective treatment is vital to improving the quality of life of patients. Along with the increase in viral pneumonia, an increasing number of traditional Chinese medicines (TCMs) have been applied to viral pneumonia treatment due to their multiple constituents, multiple targets and good safety (Wang et al., 2011; Li et al., 2015; Zhu et al., 2018; Hu et al., 2020; Li et al., 2020; Yang et al., 2020). The HouYanQing (HYQ) oral liquid used in this study contains 4 types of herbs, which have all been safely used in local traditional medicine for many years. Experimental investigations of a mouse model of pneumonia infected with influenza A virus (H1N1) indicated that HYQ exerts good therapeutic efficacy in viral pneumonia, and proteomic investigations of the molecular function of proteins perturbed by HYQ indicated that HYQ enhances the proteins involved in immune system processes, negative regulation of viral genome replication, inflammatory responses, etc. Therefore, HYQ may be a promising alternative treatment for viral pneumonia.

However, similar to many other TCMs, the ambiguous clinical effects and unclear mechanism hinder its extensive application in the clinic. In addition, rational use of this treatment in viral pneumonia is unclear. Proteomics-based investigation of signatures associated with the specific efficacy of HYQ against viral pneumonia shed light on this issue. With the aid of the discovered biomarkers, we concluded that HYQ has a specific effect on viral pneumonia such as elevated galectin-3-binding protein (*Lgals3bp*) and glutathione peroxidase 3 (*Gpx3*) levels in blood. Therefore, in this case, this treatment can be used more effectively, and the potential side effects can be minimized.

Among the signatures associated with the specific efficacy of HYQ against viral pneumonia, *Lgals3bp* promotes integrin-mediated cell adhesion and may stimulate host defense against viruses (UniProt Consortium, 2015). It was also reported that *Lgals3bp* expression is induced in viral infection and by many molecules that either mimic or are characteristic of ongoing inflammation and microbial infection, such as IFN-*α*, IFN-*β*, IFN-γ and TNF-*α*, and it has innate immune functions with special emphasis on viral and bacterial infections (Loimaranta et al., 2018). Although it was found that *Lgals3bp* may be a serological biomarker for acute dengue virus infection (Liu et al., 2016), acute hantavirus infection (Hepojoki et al., 2014), hepatitis C virus (HCV), human immunodeficiency virus (HIV) infection (Shaked et al., 2014; Yang et al., 2014; Rodríguez-Gallego et al., 2019), and hepatitis B virus-related hepatocellular carcinoma (Liu et al., 2017), the relationship between *Lgals3bp* and viral pneumonia is rarely investigated. *Gpx3* protects cells and enzymes from oxidative damage (UniProt Consortium, 2015) and may be involved in immune defense against pathogenic invasion (Bathige et al., 2015). Additionally, glutathione peroxidase 4 is reversibly induced by HCV to increase virion infectivity (Brault et al., 2016), and glutathione peroxidase activity could be an important prognostic factor in diabetic patients with Epstein-Barr virus infection (Dworzański et al., 2020) or as an adjuvant in the management of HIV-infected patients (Ogunro et al., 2006). Our present study indicates that HYQ can specifically regulate *Lgals3bp* and *Gpx3*, thus exerting a good protective effect against viral pneumonia.

In addition to the specific regulatory effect, as a multicomponent drug, HYQ can have multiple overlapping effects similar to other TCMs. As shown in the results, HYQ can also regulate intercellular cell adhesion molecule-1 (*Icam-1*), transferrin, plasma protease C1 inhibitor (*Serping1*), hemopexin (*Hpx*), protein S100-A8 (*S100a8*), and complement factor B and C3. In fact, transferrin, *Hpx*, and complement factor B and C3 have been identified by quantitative proteomics in a mouse model of influenza as potential markers of disease severity that can be clinically useful in humans (Kumar et al., 2014), and *Serping1* and complement factor B have been identified as candidate nasopharyngeal protein biomarkers in acute respiratory virus infection (Burke et al., 2017). Among these regulated molecules, *Serping1* plays a crucial role in regulating important physiological pathways, such as complement activation, and complement C3 plays a central role in the activation of the complement system (UniProt Consortium, 2015). As an essential element of the immune system, the complement system can be activated by some viruses, and a previous study indicated that *Serping1* and its network serve as important components of the innate immune system to restrict HIV-1 infection (Sanfilippo et al., 2017). *Hpx* has been proven to be a candidate marker for progression of fibrosis in HCV patients (Cheung et al., 2009) and a potential biomarker of respiratory syncytial virus-infected pneumonia (Wang et al., 2016). *S100a8* plays a prominent role in the regulation of inflammatory processes and the immune response (UniProt Consortium, 2015). After infection by some viruses, high expression of *S100a8* is observed, resulting in enhanced oxidative stress (Müller et al., 2017). Current experimental results showed that HYQ can also regulate these proteins and exert multiple effects on the complement system and inflammatory processes. Overall, HYQ will be a promising alternative for the safe and effective management of viral pneumonia.

## 5 Conclusion

In this study, a proteomics-based approach was utilized to precisely investigate the efficacy of a multicomponent traditional Chinese medicine, HouYanQing (HYQ) oral liquid, in a mouse model of pneumonia infected with influenza A virus (H1N1). Experimental results indicate that HYQ may show distinctive effects for viral pneumonia patients with the elevation of galectin-3-binding protein and glutathione peroxidase 3. This TCM also has multiple effects on the complement system and inflammatory processes. This study provides a precise investigation of the efficacy of a multicomponent drug against viral pneumonia and offers a promising alternative for personalized management of viral pneumonia.

## Data Availability

The datasets presented in this study can be found in online repositories. The names of the repository/repositories and accession number(s) can be found in the article/Supplementary Material.
