# Supplementary material for: Precise Investigation of the Efficacy of Multicomponent Drugs Against Pneumonia Infected With Influenza Virus
Source: Front Pharmacol. 2021 Nov 18;12:604009. doi: 10.3389/fphar.2021.604009 (PMC8636456; doi:10.3389/fphar.2021.604009)
Supplement: Supplementary file 2 [file DataSheet1.docx]

**Supplementary data**

1. Below is the supplementary data of the whole uncropped images of the original western blot for all replicates. All replicates of ICAM-1 and Transferrin are shown in Figure S1 and S2, and Figure S1B panel 5-8 have not been involved in the analysis. As shown in Figure S1A, panel 1-4 are the western blot results shown in Fig. 5A of the manuscript; and in Figure S1C, panel 1-4 are the western blot results shown in Fig. 5B of the manuscript. Figure S2 is the relative levels of ICAM-1 and Transferrin.


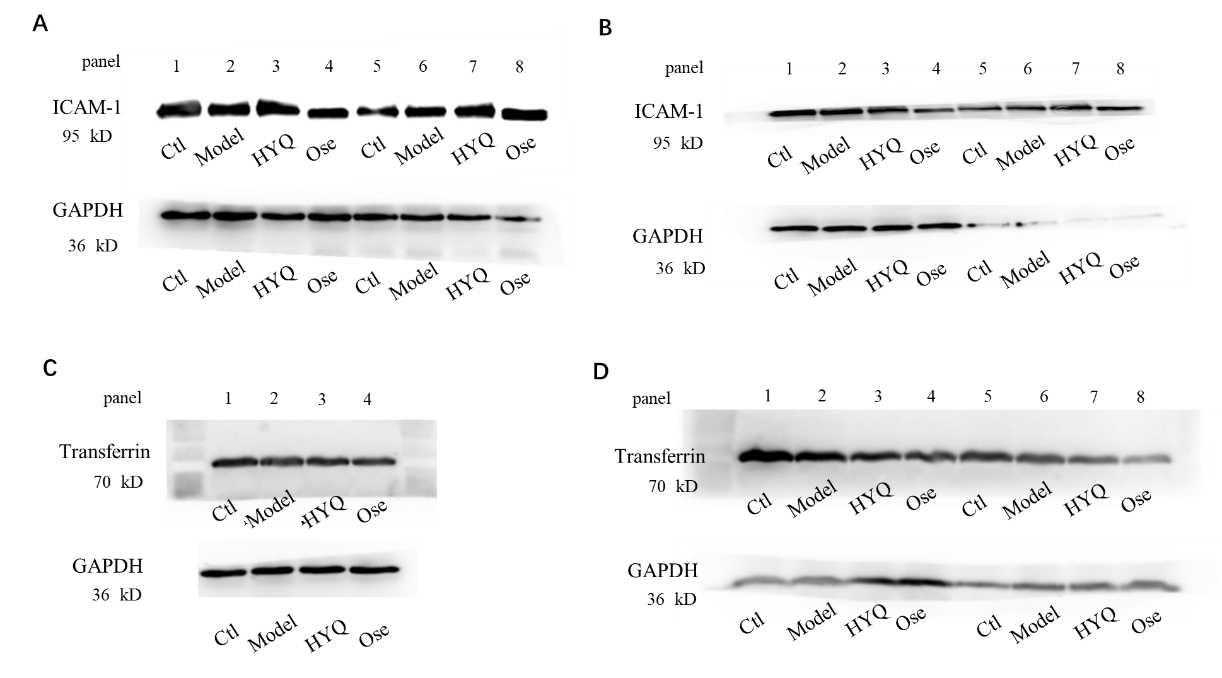


Figure S1 Uncropped images of the original western blot of ICAM-1 and transferrin.

A and B represent all replicates of ICAM-1, and C and D represent all replicates of Transferrin.


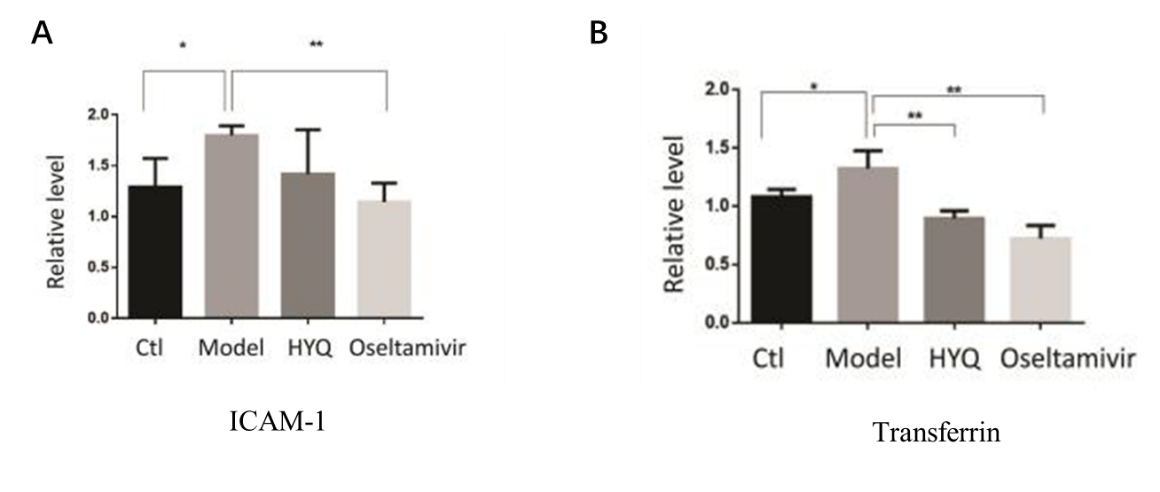


Figure S2 The relative level of ICAM-1 and Transferrin.

n = 3, * and ** represent *P*< 0.05.

1. Below is the lung tissue and pathological change at the sixth day after infection.


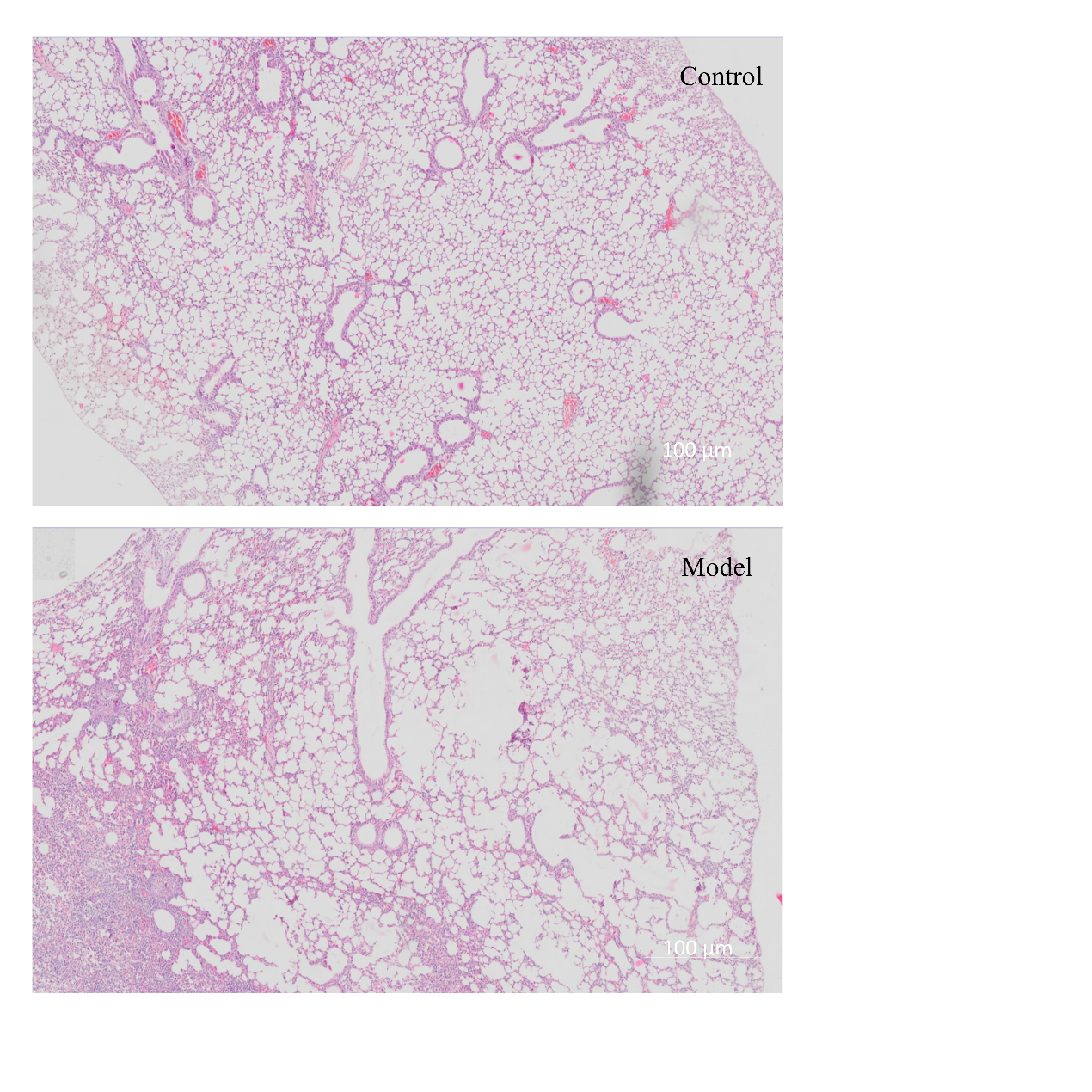


Figure S3 The lung tissue and pathological change at the sixth day after infection.

1. Below is the supplementary data of the whole uncropped images of the original western blot for all replicates of Ifitm2.

The proteins of mouse lung tissues were extracted in ice-cold RIPA lysis buffer (Solarbio, China) by ultrasound and then determined by the enhanced bicinchoninic acid protein assay kit (Thermo, USA). Thirty micrograms of each sample was loaded on 10% SDS-PAGE gels, and protein blots were transferred onto polyvinylidene fluoride membranes (Millipore, USA). After blocking with 5% nonfat milk, the blots were incubated overnight at 4 °C with the following primary antibodies: anti- interferon-induced transmembrane protein 2 (Ifitm2, Proteintech) and anti-β-actin (Proteintech). Then, the membranes were washed with a mixture of Tris-buffered saline and Tween 20 (TBST) and incubated at room temperature for 1 h with a secondary antibody conjugated to horseradish peroxidase. Finally, the protein blots were visualized using an enhanced chemiluminescence kit (Millipore, USA).


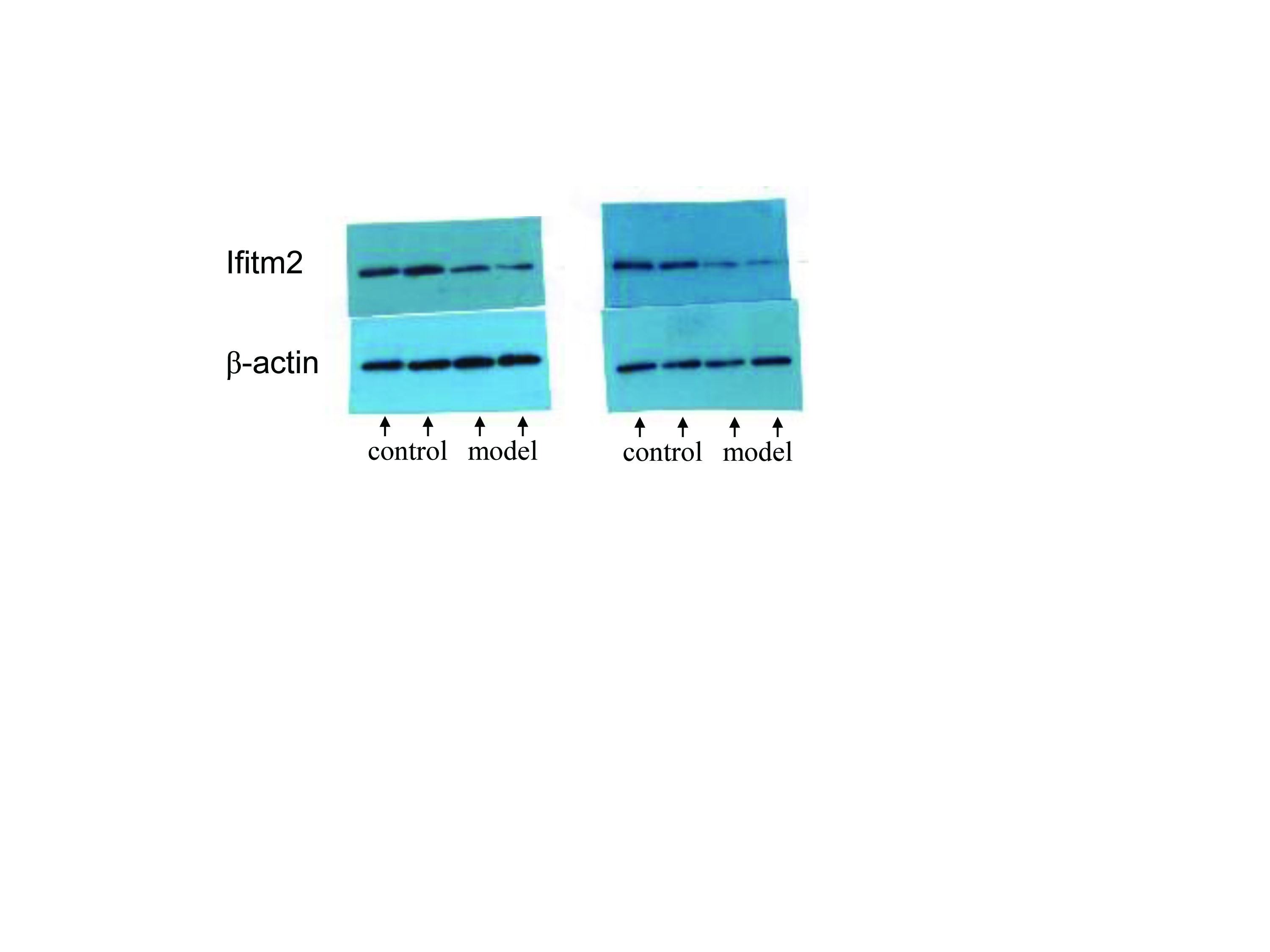


Figure S4 Uncropped images of the original western blot of Ifitm2.

1. Below is the qRT-PCR verification of the role of ICAM-l in lung tissue at the sixth day after infection and HYQ administration.

The primers of ICAM-1 for RT-PCR were as follows: upstream, 5'-TCACCGTGTATTCGTTTCCG-3'; downstream, 5'-GGCTCCGTGGTCCCCTC-3' (synthesized by Invitrogen). The primers of GAPDH for RT-PCR were as follows: upstream, 5'- GAGCCAAAAGGGTCATCATCT-3'; downstream, 5'-AGGGGCCATCCACAGTCTTC-3' (synthesized by Invitrogen). qRT-PCR was performed on an ABI 2720 Instrument (Applied Biosystems). Total RNA was extracted using TRIzol (TaKaRa, Japan) according to the manufacturer’s instructions, and reverse transcription reactions were performed using Premix Taq™ (TaKaRa, Japan). The RT-PCR protocol was as follows: 95 °C, 10 s; 60 °C, 30 s; with 40 amplification cycles. The assays were carried out in triplicate, and Ct values were calculated.

**
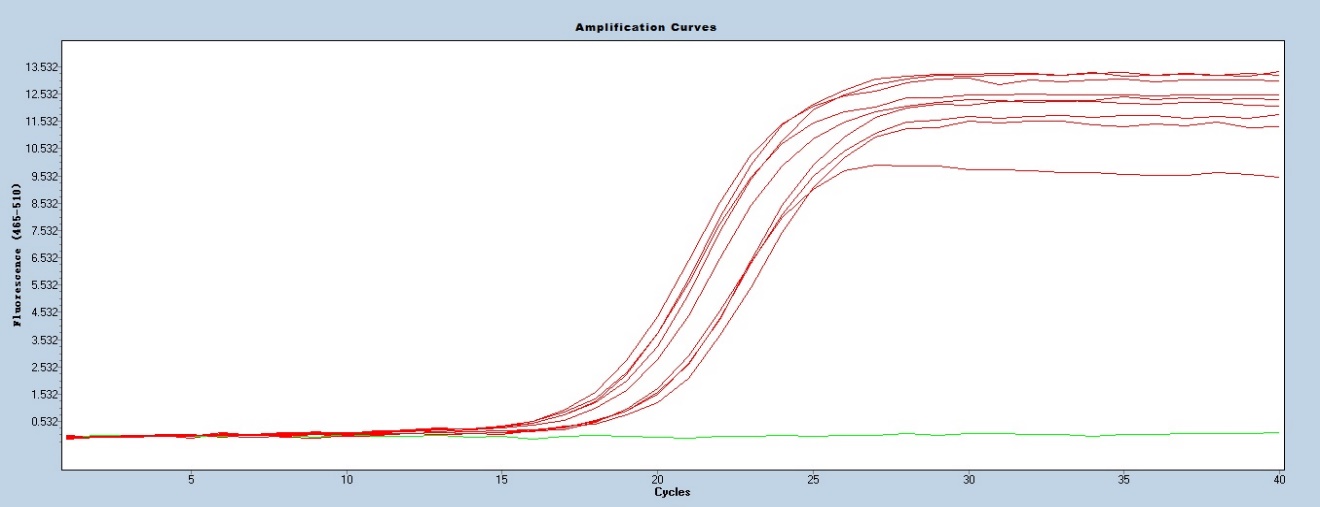
**

Figure S5 Amplification curves of GAPDH

**
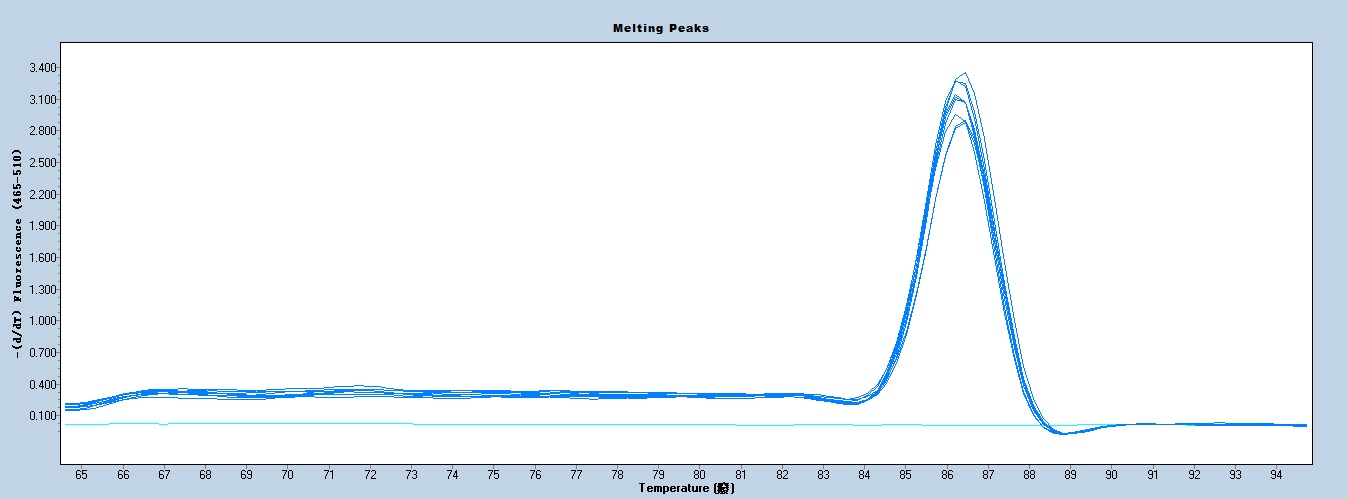
**

Figure S6 Melting peaks of GAPDH

**
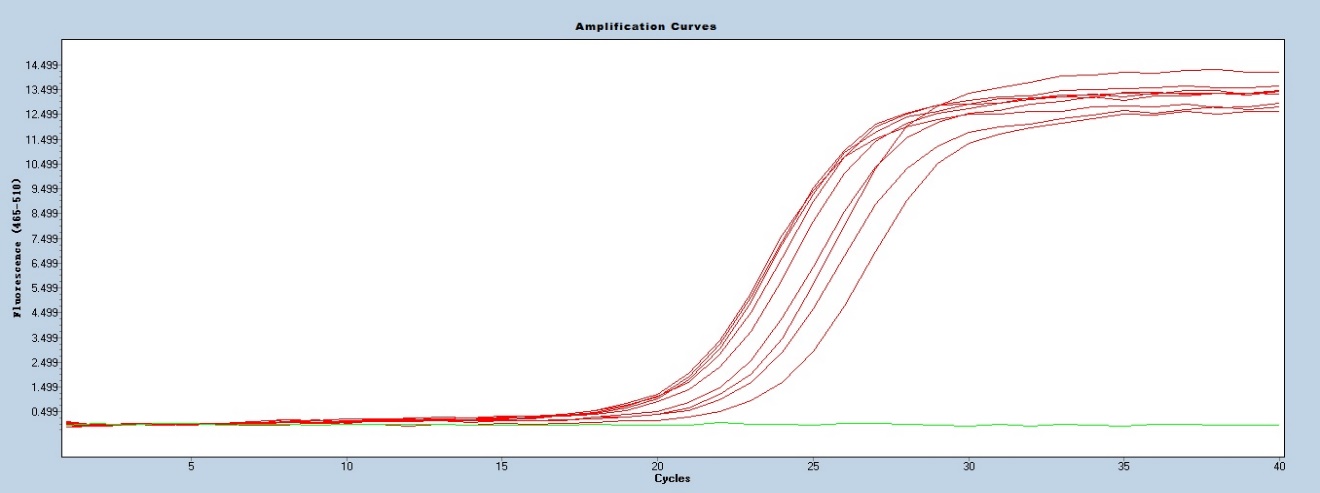
**

Figure S7 Amplification curves of ICAM-l

**
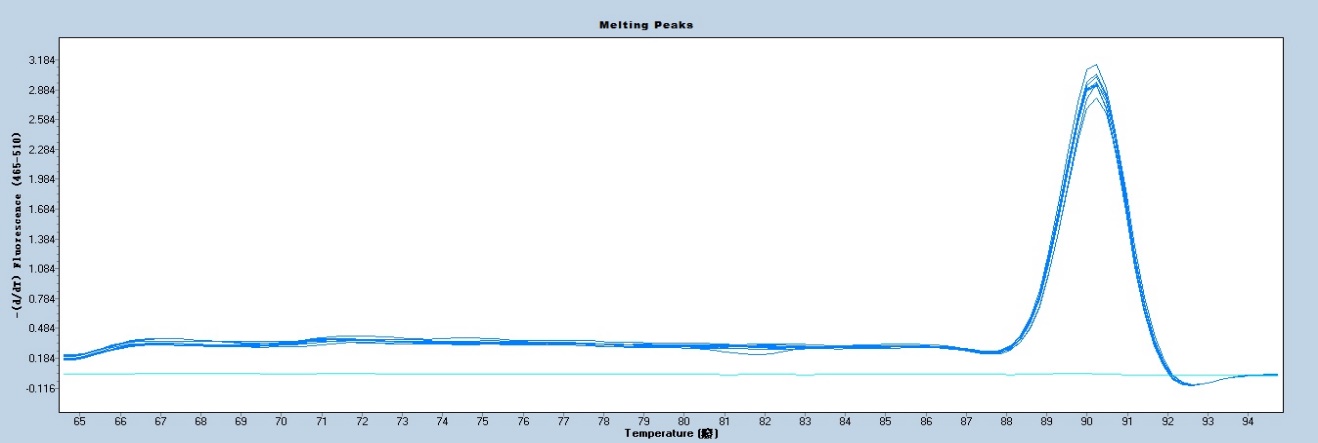
**

Figure S8 Melting peaks of ICAM-l


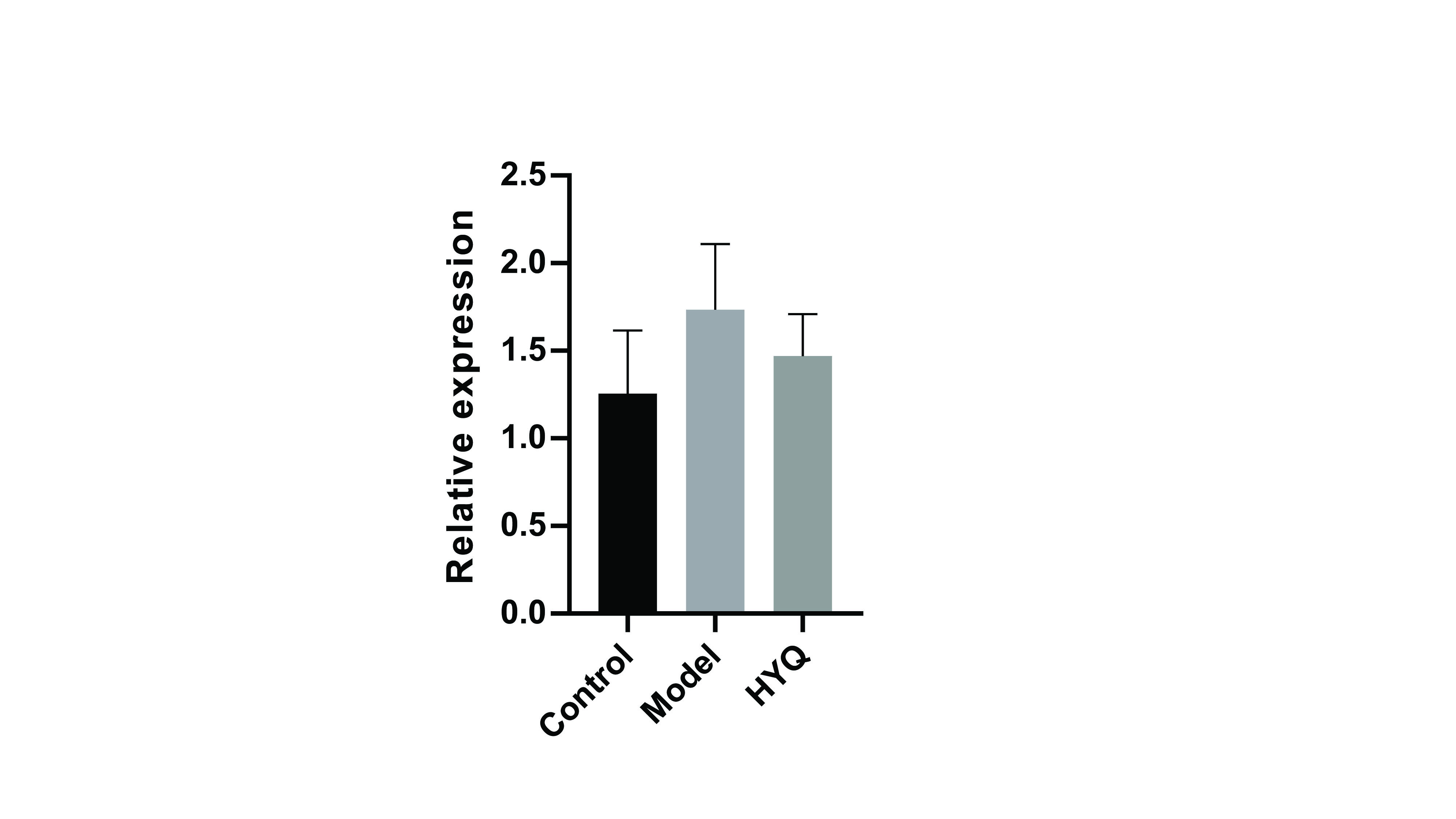


Figure S9 qRT-PCR verification of ICAM-l in lung tissue
